# NextGen microbial natural products discovery

**DOI:** 10.1111/1751-7915.12184

**Published:** 2014-12-27

**Authors:** Claudia Schmidt-Dannert

**Affiliations:** Department of Biochemistry, Molecular Biology and Biophysics, University of Minnesota1479 Gortner Avenue, St. Paul, MN, 55108, USA

Small-molecule secondary metabolites isolated from microorganisms and plants provide the chemical scaffolds of a large fraction of today's pharmaceuticals. Evolutionary forces shaped the molecular complexity of these natural products that contribute to the exquisite binding of these compounds to biological targets. Starting with the discovery of penicillin by Fleming, we have seen a rapid increase in the discovery and production of natural products and derivatives thereof as antibiotics and other drugs. But once the ‘easy to access’ bioactive compounds have been isolated, the drug discovery pipeline slowed down beginning in the 1990s. Pharmaceutical companies turned away from natural products as screening programmes led to the rediscovery of known structures and development of structurally complex natural products into drugs using synthetic methods proved to be challenging and too expensive if no reliable biological sources were available. Considering the urgent need for the development of new drugs to combat multidrug-resistant pathogens and overcome long-term side-effects and/or reduction in effectiveness of current drugs, unlocking nature's treasure trough of small-molecule chemodiversity will be crucial for next-generation drug development (Gerwick and Moore, 2012; Basmadjian *et al*., 2014; Genilloud, 2014).

Driven by advances in sequencing, gene synthesis, bioinformatics and metabolomics, the natural products discovery process is beginning to undergo a major transformation – away from the tedious isolation, screening and dereplication process to *in silico*-based bioprospecting approaches that seek to eventually transform genomic information directly into biosynthetic outputs (Lewis, 2013; Deane and Mitchell, 2014). The explosion in the number of available microbial genome sequences has given us a glance at the hidden natural product biosynthetic capacity of these organisms. Based on known sequence information for enzymes involved in synthesizing, e.g. the scaffolds of bioactive polyketides, non-ribosomal peptides or terpenes, numerous gene clusters (fungi) and operons (bacteria) can be identified in microbial genomes that are silent and for which no secondary metabolite products have been identified. This also includes many well-studied natural products producers such as *Streptomyces* and *Aspergillus* strains that express only a subset of their secondary metabolome under typical laboratory growth conditions (Brakhage, 2013; Doroghazi and Metcalf, 2013; Lim and Keller, 2013; Rebets *et al*., 2014).

Our sequencing capacity is outpacing – by orders of magnitude – our ability to identify natural products gene cluster and most importantly, translate this sequence information into screenable molecules. The number of sequenced microbial genome sequences is rapidly approaching 5000 sequenced genomes, of which a large majority is bacterial genomes with only a few hundred fungal genomes available. With this large number of sequences available, the question becomes ‘How does one most effectively search this vast sequence space for interesting natural products pathways?’ One approach commonly used is to focus on a few groups of bacteria of fungi known to produce bioactive natural products and comprehensively identify within their genomes natural products biosynthetic operons or gene cluster, and then target the most diverse biosynthetic gene cluster for characterization. In many cases, products of target gene clusters are not produced at all or only at very low levels under laboratory growth conditions, requiring gene cluster activation either through exogenous stimuli or manipulation of genetic control elements which may be strain specific and a laborious undertaking. In the case that a strain is genetically tractable, gene disruption can then be used to specifically characterize biosynthetic gene functions. This ‘reverse discovery’ approach has been quite successfully used in genome-driven bioprospecting for a number of natural products identified in bacteria and some filamentous fungi (Lewis, 2013; Deane and Mitchell, 2014; Jensen *et al*., 2014).

Such ‘reverse discovery strategies’, however, are limited to microorganisms that can be cultivated in the laboratory and that can be genetically manipulated, leaving out enormous biosynthetic diversity found in unculturable microbial species such as many higher fungi (see below) and from complex microbial ecosystems. Recent work has shown that metagenomic libraries from microbial ecosystems can be successfully arrayed and screened for large biosynthetic gene clusters of interest based on homology to conserved regions of known biosynthetic genes such non-ribosomal peptide synthases or polyketide synthases (Owen *et al*., 2013). Fungi have a tremendous capacity for natural products biosynthesis, yet only a relatively small fraction of its large biodiversity has been explored so far. Natural products pathways have mostly been characterized from a relatively small subset of Ascomycota, including filamentous fungi like *Aspergillus*, *Penicillium* and *Fusarium* that are genetically tractable and can be readily cultured in the laboratory (Lazarus *et al*., 2014). Basidiomycota, including the mushroom-forming fungi, have received almost no attention so far, despite the fact that they may have a quite distinct arsenal of natural products (Quin *et al*., 2014). Genome surveys of the few hundred genomes in Joint Genome Institute's Fungal Genomics database shows that we have barely scratched the surface of the biosynthetic potential encoded in the small number of sequences genomes that represent a minuscule fraction of the fungal diversity. Major reasons for the slow progress in characterizing the secondary metabolome of many fungi (especially many Basidiomycota) is that they are frequently hard to work with: laboratory growth may be slow or not possible and genetic tools so readily available for bacteria and filamentous fungi are largely absent.

The future of natural products and drug discovery will be greatly influenced by how quickly the scientific community can develop strategies that will enable us to move away from the slow approaches for pathway identification and characterization that depend on first the growth of the producer organism and it then being genetically tractable to some extent. Instead, we should take full advantage of rapid and affordable whole genome sequencing, RNAseq and DNA synthesis where we can move rapidly from *in silico* biosynthetic pathway identification into a high-throughput synthetic biology workflow with the concurrent analytical profiling of heterologously assembled expression libraries. Implementation of such an *in silico* to natural products discovery platform begins with the accurate identification and structural annotation of biosynthetic pathways and genes in genomic data. A number of bioinformatics tools have been developed for genomic bioprospecting (Weber, 2014), but these tools rely on algorithms trains with hidden Markov models derived from known biosynthetic genes. These models need to be expanded to capture a larger biosynthetic diversity. Coding information will then be directly used to synthesize corresponding genetic constructs suitable for high-throughput pathway assembly which could be done using already existing synthetic biology methods (Cobb *et al*., 2014).

Precise structural gene annotations will be essential for such an envisioned high-throughput synthetic biology workflow that relies on gene synthesis and assembly. From our own experience, we know that gene annotations in the genomes of many fungi are incorrect. Basidiomycota genes are very intron rich and many small intron/exons are incorrectly predicted using available models. Deep RNA sequencing of a cross-section of microbial species (fungi and bacteria) that can be grown in the lab will be crucial to develop algorithms for accurate structural annotation. High-resolution transcriptomics analysis of diverse species will enable the construction of gene co-expression networks built on physical distance to seed genes that are frequently associated with natural products biosynthetic pathways (e.g. cytochrome P450s, group transferases, transporters) could be a means for the discovery of novel pathways and sequences for broader *in silico* searchers. Considering that microbial secondary metabolite pathways are typically clustered and gene expression is co-regulated, such network analysis will be a powerful method for accurate delineation of biosynthetic gene clusters, including satellite clusters and split super-cluster pathways known in fungi. Finally, we may need to develop more than the common *Escherichia coli* and *Saccharomyces cerevisiae* host platforms for high-throughput refactoring and functional expression of pathways from a variety of sources to overcome for example potential co-factor, precursor limitations, product toxicity or the ability to express very large gene cluster. Considering the fast pace at which progress has and continues to be made in genomics and synthetic biology and also new methods being developed for compound screening and identification through high-resolution mass spectrometry (Krug and Muller, 2014), we should be optimistic that genomics-driven natural products drug discovery has bright future.
